# Supplementary material for: Pregnant Women’s Knowledge of and Attitudes towards Influenza Vaccination during the COVID-19 Pandemic in Poland
Source: Int J Environ Res Public Health. 2022 Apr 8;19(8):4504. doi: 10.3390/ijerph19084504 (PMC9031437; doi:10.3390/ijerph19084504)
Supplement: Supplementary file 1 [file ijerph-19-04504-s001.zip › File S1.pdf]

Wiedza i opinia kobiet w ciąży na temat szczepienia przeciwko grypie

Szanowne Panie,

Jesteśmy studentkami kierunku lekarskiego na Warszawskim Uniwersytecie Medycznym. W ramach działalności Studenckiego Koła Naukowego przy II Katedrze i Klinice Położnictwa i Ginekologii w Warszawie, przygotowujemy pracę naukową dotyczącą wiedzy oraz opinii kobiet w ciąży na temat szczepienia przeciwko grypie. Zawracamy się z uprzejmą prośbą o wypełnienie w pełni anonimowej ankiety, której uzyskane wyniki będą użyte tylko do analizy zbiorczej w celach naukowych. Całość zajmuje nie więcej niż 10 minut. W razie jakichkolwiek pytań lub zastrzeżeń prosimy o kontakt na praca.naukowa.wum@gmail.com

Serdecznie dziękujemy za szczere odpowiedzi i poświęcony czas.

Agnieszka Sienicka, Agata Pisula

Obraz w tle: Kobieta zdjęcie utworzone przez valuavitaly - pl.freepik.com

---

*Wymagane

1. Wiek: *

____

2. Wykształcenie: *

Zaznacz tylko jedną odpowiedź.

- ( ) Podstawowe
- ( ) Zawodowe
- ( ) Średnie
- ( ) W trakcie studiów
- ( ) Wyższe

3. Miejsce zamieszkania: *

Zaznacz tylko jedną odpowiedź.

- wieś
- miasto do 50 tysięcy mieszkańców
- miasto od 50 do 100 tysięcy mieszkańców
- miasto od 100 do 500 tysięcy mieszkańców
- miasto powyżej 500 tysięcy mieszkańców

4. Średni dochód na członka rodziny: *

Zaznacz tylko jedną odpowiedź.

- <1000zł
- 1000-2000zł
- 2000-3000zł
- 3000-4000zł
- 4000-5000zł
- 5000zł<

5. Status związku: *

Zaznacz tylko jedną odpowiedź.

- Singielka
- W związku nieformalnym
- W związku małżeńskim
- Wdowa
- Rozwiedziona

6. Religijność: *

Zaznacz tylko jedną odpowiedź.

- Wierząca
- Niewierząca

Pytania dotyczące ciąży

7. W którym tygodniu ciąży jest Pani obecnie? *

Zaznacz tylko jedną odpowiedź.

- 1-13 tydz.
- 14-27 tydz.
- 28-40 tydz.

8. Która to ciąża? *

Zaznacz tylko jedną odpowiedź.

- Pierwsza
- Druga
- Trzecia
- Czwarta lub kolejna

9. Który to będzie poród? *

Zaznacz tylko jedną odpowiedź.

- Pierwszy
- Drugi
- Trzeci
- Czwarty lub kolejny

10. Czy jest Pani w ciąży pojedynczej czy mnogiej? *

Zaznacz tylko jedną odpowiedź.

- Pojedynczej
- Mnogiej

11. Czy ma Pani dzieci? *

Zaznacz tylko jedną odpowiedź.

- Tak *Przejdź do pytania 12*
- Nie *Przejdź do pytania 14*

Jeśli ma Pani dzieci

12. W jakim wieku jest Pani dziecko/dzieci? *

Zaznacz wszystkie właściwe odpowiedzi.

- [ ] <1r.ż
- [ ] 1-3lata
- [ ] 4-6lat
- [ ] 7-15lat
- [ ] >15lat

13. Czy Pani dziecko uczęszcza/ dzieci uczęszczają do jednej z wymienionych placówek? (Żłobek, Przedszkole) *

Zaznacz tylko jedną odpowiedź.

- Tak
- Nie

Pytania dotyczące wiedzy o grypie

14. Czy wie Pani czym jest grypa? *

Zaznacz tylko jedną odpowiedź.

- ( ) Jest to infekcja wirusowa
- ( ) Jest to infekcja bakteryjna
- ( ) Jest to infekcja grzybicza
- ( ) Jest to infekcja ale nie wiem jaka
- ( ) Nie, nie wiem

15. Czy wie Pani jakie są grupy zwiększonego ryzyka ciężkiego przebiegu grypy i powikłań pogrypowych? *

Zaznacz wszystkie właściwe odpowiedzi.

- [ ] Osoby starsze
- [ ] Kobiety w ciąży
- [ ] Dzieci poniżej 5r.ż.
- [ ] Osoby z zaburzeniami odporności (m.in. osoby z niektórymi nowotworami, osoby po transplantacjach, zakażone HIV)
- [ ] Osoby z chorobami przewlekłymi (choroby płuc, serca, nerek, wątroby, metaboliczne, hematologiczne)
- [ ] Osoby ze znacznym stopniem otyłości
- [ ] Nie ma takich grup
- [ ] Nie wiem

16. Czy uważa Pani, że kobiety w ciąży są w grupie zwiększonego ryzyka ciężkiego przebiegu grypy i powikłań pogrypowych? *

Zaznacz tylko jedną odpowiedź.

- ( ) Tak
- ( ) Nie

17. Czy uważa Pani, że noworodki są w grupie zwiększonego ryzyka ciężkiego przebiegu grypy i powikłań pogrypowych? *

Zaznacz tylko jedną odpowiedź.

- Tak
- Nie

18. Czy wie Pani jakie leki są stosowane w leczeniu grypy? *

Zaznacz wszystkie właściwe odpowiedzi.

- [ ] Antybiotyki
- [ ] Leki przeciwwirusowe
- [ ] Leki przeciwbólowe, przeciwgorączkowe
- [ ] Nie ma leczenia na grypę
- [ ] Nie wiem

19. O których powikłaniach grypy w ciąży Pani słyszała? *

Zaznacz wszystkie właściwe odpowiedzi.

- [ ] Zapalenie płuc
- [ ] Ostra niewydolność oddechowa
- [ ] Ostre stany krążeniowo-oddechowe
- [ ] Tachyarytmie u płodu
- [ ] Poród przedwczesny
- [ ] Poronienie
- [ ] Obumarcie wewnątrzmaciczne płodu
- [ ] Urodzenie dziecka o niskiej masie urodzeniowej
- [ ] Nie słyszałam o żadnym

20. O których powikłaniach grypy u noworodka Pani słyszała? *

Zaznacz wszystkie właściwe odpowiedzi.

- [ ] Zapalenie płuc
- [ ] Zapalenie oskrzeli
- [ ] Niewydolność oddechowa
- [ ] Zapalenie mięśnia sercowego
- [ ] Zapalenie opon mózgowo- rdzeniowych
- [ ] Sepsa
- [ ] Nie słyszałam o żadnym

21. Czy kiedykolwiek chorowała Pani na grypę? *

Zaznacz tylko jedną odpowiedź.

- Tak
- Nie
- Nie wiem

22. Czy kiedykolwiek miała Pani robiony test na grypę? *

Zaznacz tylko jedną odpowiedź.

- Tak
- Nie
- Nie wiem

Pytania dotyczące szczepienia przeciwko grypie

23. Czy kiedykolwiek szczepiła się Pani przeciwko grypie? *

Zaznacz tylko jedną odpowiedź.

- Tak
- Nie

24. Czy w obecnej ciąży szczepiła się Pani przeciwko grypie? *

Zaznacz tylko jedną odpowiedź.

- Tak *Przejdź do pytania 33*
- Nie, ale zamierzam *Przejdź do pytania 36*
- Nie i nie zamierzam *Przejdź do pytania 35*
- Nie i nie wiem czy się zaszczepię *Przejdź do pytania 38*

25. Czy kiedykolwiek przed obecną ciążą ktoś proponował Pani szczepienie przeciwko grypie? *

Zaznacz wszystkie właściwe odpowiedzi.

- [ ] Nie
- [ ] Tak, lekarz ginekolog
- [ ] Tak, lekarz pierwszego kontaktu
- [ ] Tak, inny lekarz
- [ ] Tak, pielęgniarka/położna
- [ ] Tak, znajomi/rodzina
- [ ] Inne

26. Czy w obecnej ciąży ktoś proponował Pani szczepienie przeciwko grypie? *

Zaznacz wszystkie właściwe odpowiedzi.

- [ ] Nie
- [ ] Tak, lekarz ginekolog
- [ ] Tak, lekarz pierwszego kontaktu
- [ ] Tak, inny lekarz
- [ ] Tak, pielęgniarka/położna
- [ ] Tak, rodzina/znajomi
- [ ] Inne

27. Skąd czerpała Pani informacje na temat grypy i szczepienia przeciwko grypie? *

Zaznacz wszystkie właściwe odpowiedzi.

- [ ] Lekarz ginekolog
- [ ] Lekarz pierwszego kontaktu
- [ ] Inny lekarz
- [ ] Pielęgniarka/położna
- [ ] Rodzina/znajomi
- [ ] Media społecznościowe
- [ ] TV/Radio
- [ ] Ulotki/Kampanie/Banery
- [ ] Nie czerpałam informacji na ten temat

Inne: [ ] ____________________

28. Skąd chciałaby Pani uzyskać informacje na temat szczepienia przeciwko grypie w czasie ciąży? *

Zaznacz wszystkie właściwe odpowiedzi.

- [ ] Lekarz ginekolog
- [ ] Lekarz pierwszego kontaktu
- [ ] Inny lekarz
- [ ] Pielęgniarka/położna
- [ ] Rodzina/znajomi
- [ ] Media społecznościowe
- [ ] TV/Radio
- [ ] Ulotki/Kampanie/Banery
- [ ] Inne

Inne: [ ] ____________________

29. Czy uważa Pani, że szczepienie przeciwko grypie w czasie ciąży jest bezpieczne? *

Zaznacz tylko jedną odpowiedź.

- ( ) Nie
- ( ) Nie wiem

30. Czy wie Pani jakie są przeciwwskazania do szczepienia przeciwko grypie? *

Zaznacz wszystkie właściwe odpowiedzi.

- [ ] Ostra choroba infekcyjna przebiegająca z gorączką
- [ ] Zaostrzenia choroby przewlekłej (np. zaostrzenie astmy oskrzelowej, reumatoidalnego zapalenia stawów)
- [ ] Poważne niepożądane odczyny po poprzednim szczepieniu przeciwko grypie
- [ ] Uczulenie na białko jaja kurzego lub inne składniki szczepionki
- [ ] Nie wiem

31. Czy Pani wiedza na temat grypy oraz szczepienia przeciwko grypie jest wystarczająca do podjęcia przez Panią świadomej decyzji odnośnie tego szczepienia? *

Zaznacz tylko jedną odpowiedź.

- ( ) Tak
- ( ) Nie

32. Czy wie Pani jaki stopień odpłatności za szczepienie przeciwko grypie przysługuje kobietom w ciąży?

Zaznacz tylko jedną odpowiedź.

- ( ) Jest bezpłatne
- ( ) Jest płatne w 50%
- ( ) Jest pełnopłatne
- ( ) Nie wiem

Zaszczepiłam się

33. W którym tygodniu ciąży się Pani zaszczepiła? *

Zaznacz tylko jedną odpowiedź.

- ( ) 1-13 tydz.
- ( ) 14-27 tydz.
- ( ) 28-40 tydz.

34. Z jakiego powodu się Pani zaszczepiła? *

Zaznacz wszystkie właściwe odpowiedzi.

- [ ] W celu ochrony siebie
- [ ] W celu ochrony płodu
- [ ] Z powodu rekomendacji szczepienia przez lekarza

Inne: [ ] ______________________

Przejdź do pytania 39

Nie i nie zamierzam się szczepić

35. Dlaczego się Pani nie zaszczepiła? *

Zaznacz wszystkie właściwe odpowiedzi.

- [ ] Mam przeciwwskazania do szczepienia stwierdzone przez lekarza
- [ ] Obawiam się potencjalnych powikłań poszczepiennych
- [ ] Obawiam się wpływu szczepienia na płód
- [ ] Uważam, że przechorowanie grypy nie jest niebezpieczne
- [ ] Uważam, że istnieje niewielkie ryzyko, że zachoruję na grypę
- [ ] Unikam przyjmowania leków/produktów leczniczych w ciąży
- [ ] Uważam, że szczepienie jest mało efektywne
- [ ] Mam złe doświadczenia związane z poprzednimi szczepieniami
- [ ] Boję się igły/bólu związanego ze szczepieniem
- [ ] Nie znam placówki, w której mogłabym się zaszczepić
- [ ] Nie mam czasu
- [ ] Obawiam się kosztów szczepienia

Inne: [ ] ______________________

Przejdź do pytania 39

Nie ale zamierzam się zaszczepić

36. Dlaczego się Pani jeszcze nie zaszczepiła? *

Zaznacz wszystkie właściwe odpowiedzi.

- [ ] Lekarz zalecił późniejsze szczepienie
- [ ] Wolę poczekać ze szczepieniem do późniejszego etapu ciąży
- [ ] Miałam przeciwwskazania do szczepienia stwierdzone przez lekarza
- [ ] Obawiam się potencjalnych powikłań poszczepiennych
- [ ] Obawiam się wpływu szczepienia na płód
- [ ] Uważam, że przechorowanie grypy nie jest niebezpieczne
- [ ] Uważam, że istnieje niewielkie ryzyko, że zachoruję na grypę
- [ ] Unikam przyjmowania leków/produktów leczniczych w ciąży
- [ ] Uważam, że szczepienie jest mało efektywne
- [ ] Mam złe doświadczenia związane z poprzednimi szczepieniami
- [ ] Boję się igły/bólu związanego ze szczepieniem
- [ ] Nie znam placówki, w której mogłabym się zaszczepić
- [ ] Nie otrzymałam jeszcze wystarczających informacji dotyczących szczepienia
- [ ] Nie mam czasu
- [ ] Obawiam się kosztów szczepienia

Inne: [ ] ______________________

37. Kiedy zamierza się Pani zaszczepić? *

Zaznacz tylko jedną odpowiedź.

- ( ) W drugim trymestrze (14-27 tydz. ciąży)
- ( ) W trzecim trymestrze (28-40 tydz. ciąży)
- ( ) Po zakończeniu ciąży

Przejdź do pytania 39

Nie i nie wiem czy się zaszczepię

38. Dlaczego się Pani nie zaszczepiła? *

Zaznacz wszystkie właściwe odpowiedzi.

- [ ] Obawiam się potencjalnych powikłań poszczepiennych
- [ ] Obawiam się wpływu szczepienia na płód
- [ ] Uważam, że przechorowanie grypy nie jest niebezpieczne
- [ ] Uważam, że istnieje niewielkie ryzyko, że zachoruję na grypę
- [ ] Unikam przyjmowania leków/produktów leczniczych w ciąży
- [ ] Uważam, że szczepienie jest mało efektywne
- [ ] Mam złe doświadczenia związane z poprzednimi szczepieniami
- [ ] Boję się igły/bólu związanego ze szczepieniem
- [ ] Nie znam placówki, w której mogłabym się zaszczepić
- [ ] Nie wiedziałam, że mogę się zaszczepić
- [ ] Nie mam czasu
- [ ] Obawiam się kosztów szczepienia

Inne: [ ] ______________________________

Przejdź do pytania 39

Pytania dotyczące innych szczepień

39. Przeciwko którym z poniższych chorób zalecane są szczepienia u kobiet w ciąży? *

Zaznacz wszystkie właściwe odpowiedzi.

- [ ] Grypa
- [ ] Krztusiec
- [ ] Covid-19
- [ ] Nie wiem
- [ ] Żadne

Covid 19

40. Czy chorowała Pani na Covid-19? *

Zaznacz tylko jedną odpowiedź.

- Tak, przed ciążą
- Tak, w trakcie ciąży
- Nie

41. Czy szczepiła się Pani przeciwko Covid-19? *

Zaznacz tylko jedną odpowiedź.

- Tak, przed ciążą *Przejdź do pytania 45*
- Tak, w trakcie ciąży *Przejdź do pytania 45*
- Nie, ale zamierzam się zaszczepić *Przejdź do pytania 48*
- Nie i nie zamierzam się zaszczepić *Przejdź do pytania 47*
- Nie i nie wiem czy się zaszczepię *Przejdź do pytania 50*

42. Czy w obecnej ciąży ktoś proponował Pani szczepienie przeciwko Covid-19? *

Zaznacz wszystkie właściwe odpowiedzi.

- [ ] Nie
- [ ] Tak, lekarz ginekolog
- [ ] Tak, lekarz pierwszego kontaktu
- [ ] Tak, inny lekarz
- [ ] Tak, pielęgniarka/położna
- [ ] Tak, rodzina/znajomi
- [ ] Inne

43. Skąd chciałaby Pani uzyskać informacje na temat szczepienia przeciwko Covid-19 w czasie ciąży? *

Zaznacz wszystkie właściwe odpowiedzi.

- [ ] Lekarz ginekolog
- [ ] Lekarz pierwszego kontaktu
- [ ] Inny lekarz
- [ ] Pielęgniarka/położna
- [ ] Rodzina/znajomi
- [ ] Media społecznościowe
- [ ] TV/Radio
- [ ] Ulotki/Kampanie/Banery
- [ ] Inne

Inne: [ ] ____________________

44. Czy uważa Pani, że szczepienie przeciwko Covid-19 w czasie ciąży jest bezpieczne? *

Zaznacz tylko jedną odpowiedź.

- ( ) Tak
- ( ) Nie
- ( ) Nie wiem

Zaszczepiłam się

45. Kiedy się Pani zaszczepiła? *

Zaznacz tylko jedną odpowiedź.

- ( ) Przed ciążą
- ( ) 1-13 tydz. ciąży
- ( ) 14-27 tydz. ciąży
- ( ) 28-40 tydz. ciąży

46. Z jakiego powodu się Pani zaszczepiła? *

Zaznacz wszystkie właściwe odpowiedzi.

- [ ] W celu ochrony siebie
- [ ] W celu ochrony płodu
- [ ] Z powodu rekomendacji szczepienia przez lekarza
- [ ] W celu ułatwienia podróżowania/przemieszczania się

Inne: [ ] ______________________

Nie i nie zamierzam się zaszczepić

47. Dlaczego się Pani nie zaszczepiła? *

Zaznacz wszystkie właściwe odpowiedzi.

- [ ] Mam przeciwwskazania do szczepienia stwierdzone przez lekarza
- [ ] Obawiam się potencjalnych powikłań poszczepiennych
- [ ] Obawiam się wpływu szczepienia na płód
- [ ] Uważam, że przechorowanie Covid-19 nie jest niebezpieczne
- [ ] Uważam, że istnieje niewielkie ryzyko, że zachoruję na Covid-19
- [ ] Unikam przyjmowania leków/produktów leczniczych w ciąży
- [ ] Uważam, że szczepienie jest mało efektywne
- [ ] Mam złe doświadczenia związane z poprzednimi szczepieniami
- [ ] Boję się igły/bólu związanego ze szczepieniem
- [ ] Nie znam placówki, w której mogłabym się zaszczepić
- [ ] Nie mam czasu
- [ ] Obawiam się kosztów szczepienia

Inne: [ ] ______________________

Nie ale zamierzam się zaszczepić

48. Dlaczego się Pani jeszcze nie zaszczepiła? *

Zaznacz wszystkie właściwe odpowiedzi.

- [ ] Lekarz zalecił późniejsze szczepienie
- [ ] Wolę poczekać do późniejszego etapu ciąży
- [ ] Miałam przeciwwskazania do szczepienia stwierdzone przez lekarza
- [ ] Obawiam się potencjalnych powikłań poszczepiennych
- [ ] Obawiam się wpływu szczepienia na płód
- [ ] Uważam, że przechorowanie Covid-19 nie jest niebezpieczne
- [ ] Uważam, że istnieje niewielkie ryzyko, że zachoruję na Covid-19
- [ ] Unikam przyjmowania leków/produktów leczniczych w ciąży
- [ ] Uważam że szczepienie jest mało efektywne
- [ ] Mam złe doświadczenia związane z poprzednimi szczepieniami
- [ ] Boję się igły/bólu związanego ze szczepieniem
- [ ] Nie znam placówki, w której mogłabym się zaszczepić
- [ ] Nie otrzymałam jeszcze wystarczających informacji dotyczących szczepienia
- [ ] Nie mam czasu
- [ ] Obawiam się kosztów szczepienia

Inne: [ ] ____________________

49. Kiedy zamierza się Pani zaszczepić? *

Zaznacz tylko jedną odpowiedź.

- ( ) W drugim trymestrze (14-27 tydz. ciąży)
- ( ) W trzecim trymestrze (28-40 tydz. ciąży)
- ( ) Po zakończeniu ciąży

Nie i nie wiem czy się zaszczepię

50. Dlaczego się Pani nie zaszczepiła? *

Zaznacz wszystkie właściwe odpowiedzi.

- [ ] Obawiam się potencjalnych powikłań poszczepiennych
- [ ] Obawiam się wpływu szczepienia na płód
- [ ] Uważam, że przechorowanie Covid-19 nie jest niebezpieczne
- [ ] Uważam, że istnieje niewielkie ryzyko, że zachoruję na Covid-19
- [ ] Unikam przyjmowania leków/produktów leczniczych w ciąży
- [ ] Uważam, że szczepienie jest mało efektywne
- [ ] Mam złe doświadczenia związane z poprzednimi szczepieniami
- [ ] Boję się igły/bólu związanego ze szczepieniem
- [ ] Nie znam placówki, w której mogłabym się zaszczepić
- [ ] Nie wiedziałam, że mogę się zaszczepić
- [ ] Nie mam czasu
- [ ] Obawiam się kosztów szczepienia

Inne: [ ] ______________________

Ta treść nie została utworzona ani zatwierdzona przez Google.

Formularze Google
