# Supplementary material for: Pregnant Women’s Knowledge of and Attitudes towards Influenza Vaccination during the COVID-19 Pandemic in Poland
Source: Int J Environ Res Public Health. 2022 Apr 8;19(8):4504. doi: 10.3390/ijerph19084504 (PMC9031437; doi:10.3390/ijerph19084504)
Supplement: Supplementary file 1 [file ijerph-19-04504-s001.zip › Table S1.pdf]

Participants' vaccination status depending on the sociodemographic characteristics.

		Ever been vaccinated			
**Category (n=515)**	**Variables**	**Yes**		**No**	
		**Frequency**	**Percentage**	**Frequency**	**Percentage**
**Age**	19-25	19	29.7	45	70.3
	26-30	93	41.7	130	58.3
	31-35	76	46.3	88	53.7
	36-40	32	56.1	25	43.9
	41-43	4	57.1	3	42.9
**Education**	Primary	0	0.0	2	100.0
	Vocational	2	40.0	3	60.0
	Secondary	17	28.8	42	71.2
	University students	8	44.4	10	55.6
	Higher	197	45.7	234	54.3
**Average income per household member**	< 1000 PLN	3	20.0	12	80.0
	1000 – 2000 PLN	15	23.1	50	76.9
	2000 – 3000 PLN	40	37.0	68	63.0
	3000 – 4000 PLN	66	50.0	66	50.0
	4000 - 5000 PLN	36	45.6	43	54.4
	> 5000PLN	64	55.2	52	44.8
**Place of residence**	Countryside	37	35.6	67	64.4
	Small village (<50k residents)	30	44.1	38	55.9
	Town (50k – 100k residents)	9	26.5	25	73.5
	City (100k-500k)	42	41.6	59	58.4
	City (> 500k)	106	51.0	102	49.0
**Current relationship status**	Single	2	66.7	1	33.3
	Informal relationship	39	40.6	57	59.4
	Married	183	44.2	231	55.8
	Divorced	0	0.0	2	100.0
