# Supplementary material for: Pregnant Women’s Knowledge of and Attitudes towards Influenza Vaccination during the COVID-19 Pandemic in Poland
Source: Int J Environ Res Public Health. 2022 Apr 8;19(8):4504. doi: 10.3390/ijerph19084504 (PMC9031437; doi:10.3390/ijerph19084504)
Supplement: Supplementary file 1 [file ijerph-19-04504-s001.zip › Table S2.pdf]

Participant's vacciantion status during current pregnancy depending on the sociodemographic status

Category (n=515)		Been vaccinated against influenza during current pregnancy							
		Yes		No, but I am going to		No and I am not going to		No and I do not know if I will get vaccinated	
		n	%	n	%	n	%	n	%
Age	19-25	13	20.3	4	6.3	34	53.1	13	20.3
	26-30	45	20.2	40	17.9	98	43.9	40	17.9
	31-35	35	21.3	37	22.6	72	43.9	20	12.2
	36-40	13	22.8	9	15.8	30	52.6	5	8.8
	41-43	2	28.6	0	0.0	4	57.1	1	14.3
Education	Primary	0	0.0	0	0.0	1	50.0	1	50.0
	Vocational	1	20.0	1	20.0	2	40.0	1	20.0
	Secondary	8	13.6	7	11.9	30	50.8	14	23.7
	University students	3	16.7	3	16.7	9	50.0	3	16.7
	Higher	96	22.3	79	18.3	196	45.5	60	13.9
Average income per household member	< 1000 PLN	1	6.7	2	13.3	7	46.7	5	33.3
	1000 – 2000 PLN	5	7.7	5	7.7	47	72.3	8	12.3
	2000 – 3000 PLN	18	16.7	13	12.0	55	50.9	22	20.4
	3000 – 4000 PLN	30	22.7	26	19.7	54	40.9	22	16.7
	4000 - 5000 PLN	18	22.8	16	20.3	35	44.3	10	12.7
	> 5000PLN	36	31.0	28	24.1	40	34.5	12	10.3
Place of residence	Countryside	17	16.3	12	11.5	56	53.8	19	18.3
	Small village (<50k residents)	14	20.6	9	13.2	35	51.5	10	14.7
	Town (50k – 100k residents)	1	2.9	1	2.9	25	73.5	7	20.6
	City (100k-500k)	19	18.8	14	13.9	50	49.5	18	17.8
	City (> 500k)	57	27.4	54	26.0	72	34.6	25	12.0
Current relationship status	Single	1	33.3	1	33.3	1	33.3	0	0.0
	Informal relationship	17	17.7	20	20.8	40	41.7	19	19.8
	Married	90	21.7	69	16.7	195	47.1	60	14.5
	Divorced	0	0.0	0	0.0	2	100.0	0	0.0
