# Supplementary material for: Pregnant Women’s Knowledge of and Attitudes towards Influenza Vaccination during the COVID-19 Pandemic in Poland
Source: Int J Environ Res Public Health. 2022 Apr 8;19(8):4504. doi: 10.3390/ijerph19084504 (PMC9031437; doi:10.3390/ijerph19084504)
Supplement: Supplementary file 1 [file ijerph-19-04504-s001.zip › Table S3.pdf]

Participant's vacciantion status against COVID-19 depending on the sociodemographic characteristics

Category (n=515)		Been vaccinated against COVID-19									
		Yes, before pregnancy		Yes, during pregnancy		No, but I am going to		No and I am not going to		No and I do not know if I will get vaccinated	
		n	%	n	%	n	%	n	%	n	%
Age	19-25	17	26.6	13	20.3	3	4.7	22	34.4	9	14.1
	26-30	73	32.7	63	28.3	16	7.2	43	19.3	28	12.6
	31-35	50	30.5	50	30.5	8	4.9	38	23.2	18	11.0
	36-40	14	24.6	16	28.1	3	5.3	15	26.3	9	15.8
	41-43	0	0.0	3	42.9	2	28.6	2	28.6	0	0.0
Education	Primary	0	0.0	0	0.0	0	0.0	2	100.0	0	0.0
	Vocational	1	20.0	1	20.0	0	0.0	1	20.0	2	40.0
	Secondary	10	16.9	11	18.6	3	5.1	21	35.6	14	23.7
	University students	4	22.2	6	33.3	0	0.0	6	33.3	2	11.1
	Higher	139	32.3	127	29.5	29	6.7	90	20.9	46	10.7
Average income per household member	< 1000 PLN	1	6.7	2	13.3	1	6.7	6	40.0	5	33.3
	1000 – 2000 PLN	6	9.2	13	20.0	4	6.2	29	44.6	13	20.0
	2000 – 3000 PLN	31	28.7	23	21.3	6	5.6	29	26.9	19	17.6
	3000 – 4000 PLN	45	34.1	38	28.8	10	7.6	24	18.2	15	11.4
	4000 - 5000 PLN	30	38.0	23	29.1	6	7.6	14	17.7	6	7.6
	> 5000PLN	41	35.3	46	39.7	5	4.3	18	15.5	6	5.2
Place of residence	Countryside	15	14.4	29	27.9	5	4.8	37	35.6	18	17.3
	Small village (<50k residents)	23	33.8	10	14.7	6	8.8	18	26.5	11	16.2
	Town (50k – 100k residents)	4	11.8	7	20.6	6	17.6	14	41.2	3	8.8
	City (100k-500k)	31	30.7	27	26.7	8	7.9	21	20.8	14	13.9
	City (> 500k)	81	38.9	72	34.6	7	3.4	30	14.4	18	8.7
Current relationship status	Single	2	66.7	0	0.0	0	0.0	1	33.3	0	0.0
	Informal relationship	29	30.2	27	28.1	7	7.3	20	20.8	13	13.5
	Married	123	29.7	118	28.5	25	6.0	98	23.7	50	12.1
	Divorced	0	0.0	0	0.0	0	0.0	1	50.0	1	50.0
